# Indirect Costs of Rheumatoid Arthritis Depending on Type of Treatment—A Systematic Literature Review

**DOI:** 10.3390/ijerph16162966

**Published:** 2019-08-17

**Authors:** Bogdan Batko, Paulina Rolska-Wójcik, Magdalena Władysiuk

**Affiliations:** 1 Department of Rheumatology, J. Dietl Specialist Hospital, Skarbowa 1 St, 31-121 Cracow, Poland; 2 HTA Consulting, Starowislna 17/3 St, 31-038 Cracow, Poland

**Keywords:** rheumatoid arthritis, productivity loss, workplace, absenteeism, cost of illness, economic burden, indirect cost, presenteeism, sick leave, systematic review

## Abstract

The economic burden of rheumatoid arthritis (RA) on society is high. Disease-modifying antirheumatic drugs (DMARDs) are the cornerstone of therapy. Biological DMARDs are reported to prevent disability and improve quality of life, thus reducing indirect RA costs. We systematically reviewed studies on the relationship between RA and indirect costs comparing biological treatment with standard care. Studies, economic analyses, and systematic reviews published until October 2018 through a MEDLINE search were included. A total of 153 non-duplicate citations were identified, 92 (60%) were excluded as they did not meet pre-defined inclusion criteria. Sixty-one articles were included, 17 of them (28%) were reviews. After full-text review, 28 articles were included, 11 of them were reviews. Costs associated with productivity loss are substantial; in several cases, they may represent over 50% of the total. The most common method of estimation is the Human Capital method. However, certain heterogeneity is observed in the method of estimating, as well as in the resultant figures. Data from included trials indicate that biological therapy is associated with improved labor force participation despite an illness, in which the natural course of disease is defined by progressive work impairment. Use of biological DMARDs may lead to significant indirect cost benefits to society.

## 1. Introduction

Rheumatoid arthritis (RA) is a progressive, chronic autoimmune disease that carries a significant global burden and affects economic activity [1,2]. Progressive joint damage, pain, disability, and premature mortality are hallmarks of RA, particularly if not treated early and appropriately [3]. Environmental and socioeconomic factors may also influence disease status [4]. Patients often suffer from symptoms that may be objectively difficult to quantify (e.g., fatigue), while they impact work performance, and are often disregarded by employers [5]. Close to a third of patients may be permanently work disabled within the first three years of disease, which leads to both societal and individual costs [6]. In 1992, health and social care direct costs were estimated at over £600 million in the United Kingdom (UK), with indirect costs (lost productivity) estimated at an additional £651million [7]. An estimate of the annual economic burden of RA in the United States (US) puts the societal impact at $19.3 billion (in 2005), with 56% ($10.9 billion) due to indirect costs [8]. The introduction of new treatment strategies and biological agents in the last 20 years, for which clinical results indicate declining physical disability rates, is revolutionary [9]. The economic question long debated is whether greater efficacy justifies its higher costs [10]. With increased understanding of pathogenesis, novel therapies are emerging, however, many are still limited to experimental settings, and conventional synthetic disease modifying anti-rheumatic drugs along with biological agents remain the mainstay of therapy [11]. In this respect, it is worth mentioning that people with RA are recommended to use a wide variety of self-management methods. The purpose of implementing these programs is reducing pain and inflammation, reducing the risk of deformities developing, and maintaining or improving function [12].

Costs of illness can usually be divided into the more tangible direct costs, e.g., healthcare expenditures associated with treatment and management, and indirect costs related to productivity loss [13]. The latter can be valued through either the human capital method (HCM) or friction cost method (FCM) [14]. HCM is based on a societal perspective of future productivity, in which an individual’s lack of contribution to society is projected, carrying the assumption of employee irreplaceability [13]. Conversely, the FCM assesses productivity loss until an individual is replaced [15]. High disease-related costs of RA for the individual, healthcare system and society have been previously assessed by cost-of-illness studies in numerous countries [16,17]. Studies have shown that presenteeism reduces work productivity to a greater extent than absenteeism, and is considered the largest component of work productivity loss [18,19]. In chronic conditions, including arthritis, presenteeism is regarded as a major contributor to costs [19,20,21]. Comprehensive data on the impact of biological agents on indirect costs are limited. Previous estimates may not be relevant to the current era of biological therapy. 

This literature review examines the relationship between the economic aspects of biological therapy on absenteeism, and on presenteeism, and it provides an overview of indirect costs of absenteeism and presenteeism in RA patients treated with biological therapies. It should be noted that our search strategy is prone to a degree of bias, owing to the adoption of several brand names for biological agents, which may lead to incomplete retrieval of records, as e.g., some agents may be restricted in certain countries.

## 2. Materials and Methods 

**Figure 1:**
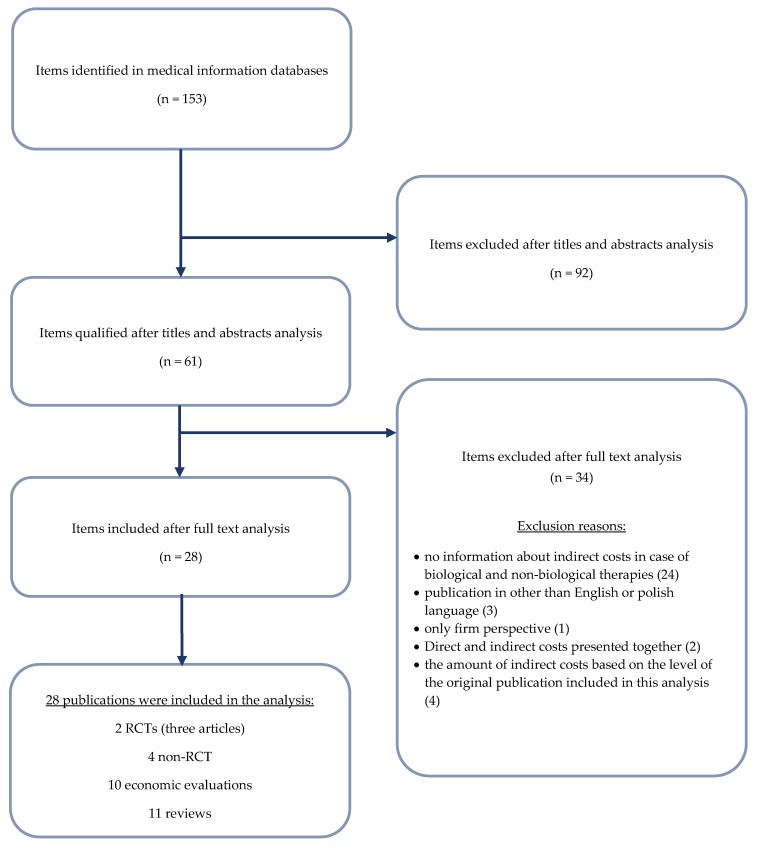
Scheme of selection of clinical trials found in the systematic review in accordance with PRISMA.

We performed a systematic literature review aimed to identify studies or economic evaluations relevant to indirect costs in RA patients treated with biological agents (Table A1). Studies, analyses, and reviews on RA economic topics published until October 2018 through MEDLINE literature database search were examined. Search results were screened in title relevance and full-text copies were retrieved if a publication was an article, which fulfilled the following criteria: (i) subjects at or over the age of 18, with a diagnosis of RA, (ii) a comparison of any type of biological therapy with any kind of treatment was performed, (iii) a measure of productivity loss was a study outcome, (iv) or the article was a narrative or systematic review of such studies, and (v) it was in English language. In the search strategy the authors adopted several brand names for biological agents. We excluded duplicate reports. This process was conducted by two readers and is summarized in Figure 1. Studies were evaluated in a three-step process; title list consideration, evaluation of abstracts that passed the latter step and finally, articles that were of relevance were reviewed. Disagreement between the two reviewers was resolved by consensus of a third party. Data from eligible primary studies of indirect RA costs were extracted into abstraction forms and a spreadsheet was used for data entry. Productivity was investigated with a focus on two different measures: presenteeism, which is synonymous with work limitation, and absenteeism, or time off work due to RA [18].

We screened 153 non-duplicate citations (last update: 2 October 2018), 92 of which (60%) were excluded as they did not meet pre-defined inclusion criteria. Sixty-one articles were included, 17 of which (28%) were reviews. After full-text review, 28 articles were included, 11 of them were reviews. Exclusion reasons: no information about indirect costs in case of biological and non-biological therapies (24), publication in Chinese language (3), only company perspective (1), direct and indirect costs presented together (2), the amount of indirect costs based on the level of the original publication included in this analysis (4). The selection process is presented in the PRISMA diagram (Figure 1).

## 3. Results

Two of identified studies were randomized controlled trials (RCT) (three articles) [22,23,24], four were observational studies/registry or cross-sectional in design [25,26,27,28], and 10 of them were economic evaluations [29,30,31,32,33,34,35,36,37,38]. Eleven reviews were included in the review and described in the discussion section [14,24,38,39,40,41,42,43,44,45,46]. Risk of bias in RCTs and observational trials included in systematic review were assessed (details in appendix) using the modified Cochrane Collaboration tool to assess risk of bias for randomized controlled trials and National Institute for Health and Care Excellence (NICE) scale to assess the risk of bias in observational trials [47,48].

Most of studies estimated the indirect costs using the human capital method [23,24,26,28], two of them the friction cost method [22,27], and one of them did not report the method [25]. Measures of work productivity loss (while at work) due to presenteeism were the Work Limitations Questionnaire (WLQ) [23], the Work Productivity and Activity Impairment Questionnaire (WPAI) [23], the Work Ability Index (WAI), and Health and Labor Questionnaire (HLQ) [24]. Although all measures were developed to measure presenteeism, they vary in construct, recall period, attribution, and reference frame, as previously discussed [46]. Indirect costs due to absenteeism are often calculated as the number of hours or days absent multiplied by average pay rate [23], with HCM being the preferred method. As actual salary data are not always available, the mean salaries of corporations, and mean or median wages (overall or by age and gender) are often used as a surrogate for actual wages. In included studies the source for productivity loss information was patient self-report [23]. Data were collected via a questionnaire completed at the clinic [23]. With few exceptions, the patients were identified via a physician diagnosis of RA, usually according to American College of Rheumatology or American Rheumatology Association criteria (ACR) [22,23,24]. Functional disability was measured according to the scale of Health Assessment Questionnaire (HAQ) [22,23,24]. 

Evidence from studies in the literature suggests that treatment with the tumor necrosis factor (TNF) inhibitors etanercept [23,25,26], adalimumab [24,25,28], and infliximab (IFX) [22,25] increases productivity, and reduces the degree of RA-related absenteeism and presenteeism. 

### 3.1. Absenteeism and Presenteeism

For the studies fulfilling the criteria of our review we provide an overview of design, patient population, and comparator (Table 1), and outcome measures for indirect costs with respective monetary gains associated with improved absenteeism (Table 1 and Table 2) and presenteeism (Table 1 and Table 3). Risk of bias was also assessed (Table A2 and Table A3). Studies are also summarized below. 

The COMET (COmbination of Methotrexate and ETanercept) trial was a 2-year double-blind randomized clinical trial. In a sub-analysis provided by Anis et al., the impact of etanercept (ETA) addition to methotrexate (MTX) on productivity outcomes was evaluated during the first year in MTX naïve, early RA patients. Absenteeism was an outcome, assessed through the number of stopped and missed work days, and reduced working time in patients who were working at baseline, as reported by them in questionnaires. Outcomes were recorded at four visits during follow-up, but patients in part/fulltime work with at least one visit were included. The recall period was 4 and 8 weeks between the first two and latter two visits. Due to a lack of exact data for the dates of work stoppage/renewal, two scenarios for maximum and minimum absenteeism were constructed, however, they remain reliant on some assumptions. Patients treated with ETA + MTX vs. MTX were reported with 18 fewer missed work days (95% CI: 2, 34). Total absenteeism was significantly improved under both scenarios, with 37 fewer work days lost at maximum (95% CI: 6; 68), and 22 for minimum scenario (95% CI: 2; 43). Presenteeism was not directly assessed in the COMET trial and had to be indirectly imputed from a sensitivity analysis for base estimates for absenteeism. Potential limitations in the calculation of presenteeism have to be addressed; presenteeism estimates may vary with part-time workers who may devote more time to self-care, while the method of presenteeism imputation from absenteeism may result in collinearity and inflate the analyses. Independent of chosen measure, productivity loss measures in MTX-treated patients were generally higher than with combination therapy. However, the magnitude seems to depend on the mode of measure [23].

Allaart et al. investigated the findings of BeSt trial, in which four treatment strategies were evaluated (Table 1). Patients in groups 3 and 4 were observed with faster improvement in utility measures, though all strategies were comparable at 2 years. In the proportion of patients with initial combination with MTX and IFX, an improvement of four absent hours/week when compared to group 3 was observed at 2 years. It was concluded that quality of life shares a relationship with productivity; a decrease in 0.1 on utility was associated with two less working hours per week [22]. Some doubts have been raised over BeSt trial analyses over baseline differences in productivity [49]. In the BeSt Study utilizing the FCM, overall societal costs were estimated at €19,905 (seq. monotherapy), €15,926 (step-up comb. therapy), €17,810 (initial comb. therapy), and €28,547 (MTX + INF) (*p* ≤ 0.05 Group 4 vs. Groups 1–3), but total medical costs were estimated at €10,792, €7288, €7809, and €23,761, respectively, in Groups 1–4. On a side note, 59% of patients did not have a job, while cost-effectiveness was only reported for the entire group [22].

Zhang et al. evaluated adalimumab (ADA) and productivity measures at baseline and following 12 weeks of treatment in RA of moderate to severe character. Work productivity outcomes and associated costs were assessed through the health labor questionnaire (HLQ). This entailed a sub-study of the Canadian Adalimumab Clinical Trial (CanAct), in which the economic burden of work productivity loss was determined from a societal perspective. A comparison with regard to treatment response status was performed. No significant effect on employment was observed in short-term, though improvement in absenteeism (0.5 work days per 2 weeks) and unpaid work productivity (3.5 fewer hours unpaid help per 2 weeks) was observed after 12 weeks. Clinical response was associated with improvements [24].

Klimes et al. performed a cost-of-illness investigation, which included productivity costs for consecutive RA outpatients of productive age. Patients were divided by HAQ scores into groups. Unemployed patients, retired pensioners, and students were excluded. Sick leave days and time on disability pension, both within a maximum friction period, were obtained by providers at month 0, when functional scores were determined. It was observed that higher HAQ scores are linked to downward trends of sick leave, which may be explained through work status. Higher HAQ scores are associated with an increased proportion of patients on disability pensions. When examining overall annual costs, biological treatment was associated with greater mean (SD) productivity costs of 2090 (1888), while patients without have 1304 (1830) respectively. However, these groups differed in demographic and disease characteristics. Annual mean total (direct and productivity) costs per patient treated with biologicals, without biological treatment, and from the overall cohort were €14,763, €3559, and €8882, respectively [27]. 

Augustsson et al. reported data from the prospective STURE register, and analyzed the relationship of ETA, infliximab (IFX), and ADA with workforce participation in over 5 years of follow-up. Subjects not participating in workforce, old age and permanent work disability pensioners, and over 55 years of age were not included, while only first treatment period data was considered. The data for employment outcome measures were obtained from questionnaires. Some data was missing, and sample size decreased due to treatment (discontinuation) and management (irregular follow-up) related factors. Conclusions of the associated gains include more hours worked/week in year 1 and annual improvements in years 2–5 by using TNF-antagonist. Economic gains were projected as close to 40% of annual anti-TNF drug cost when treatment is continued. However, there was no control group and comparison was based on an assumption that no loss in productivity occurs over 5 years without anti-TNF agents. Potential sources of bias include questionnaire data based on self-reported claims and differential dropout of patients, which limits these findings to patients who continue TNF inhibitors [25]. 

Hone et al. reported data from an observational, longitudinal study of part/fulltime employed patients with moderate to severe RA commencing ETA. Work productivity and activity impairment questionnaire (WPAI) was administered via telephone communication, while medical chart review was performed only at 6-month timepoint completion. After 6 months, mean (SD) change from baseline for absenteeism was −0.5 (17.0); 95%CI −6.1, −1.0 for all patients, −4.1 (14.2); 95% CI −6.5, −1.8 for continuers and insignificant −1.0 (25.9); 95% CI −10.3, −8.4 for discontinuers [26]. Annual economic gains in productivity could compensate the cost of ETA in part or total. Potential bias may be associated with an overestimation through WPAI, as compared to other measuring tools [50], or questionnaire design with attribution and responder bias.

Tanaka et al. published a report from the ANOUVEAU cohort, which was a 48 week observational, multicenter study of ADA among inadequate responders to conventional treatment. Productivity outcomes, including absenteeism and presenteeism, were assessed through validated questionnaires (WPAI/RA), and were significantly improved by administrating ADA. This was observed for different employment status (paid workers, part time, and home makers) when comparing to baseline [28]. Potential limitations include a single study arm, and a comparison of outcomes from baseline. 

### 3.2. Overview of Indirect Costs

In the COMET trial, total work productivity loss (the sum of absenteeism and presenteeism) was 42 work days or £2968 less in the ETA + MTX treatment group compared to MTX under Scenario I, or 31 work days or £2212 less under Scenario II (when the WPAI was used to estimate the percentage work productivity loss at work, total work productivity loss). When WLQ was used to estimate the percentage work productivity loss at work, total work productivity loss was 37 work days or £2607 less in the ETA + MTX group than MTX group under Scenario I, or 23 work days or £1646 less under Scenario II [23]. 

In the CanAct clinical trial, costs saved by responders were up to CAN$155.04 per 2 weeks greater than in nonresponses. In a study by Zhang et al. four measures of presenteeism were compared in patients with osteoarthritis and RA including the HLQ, WLQ, HPQ, and the WPAI. Based on a 2-week follow-up period, the average (SD) number of hours lost was conversion into CAN$ resulted in average indirect costs over a 2-week period of, respectively, CAN$30.03, $83.05, $284.07, and $285.10 [24].

Hone et al. projected 12-month gain in work productivity for continuers of ETA at 284.5 h per patient, equating to $3233–22,533 relative to the annual income level. In these employed patients with moderate to severe RA, etanercept significantly decreases overall work and activity impairment, which may compensate a proportion, if not the full cost, associated with treatment ($20,190) [26].

Reports from the ANOUVEAU registry on RA-related productivity loss of Japanese society estimate the impactat $9.80 billion. Annual decrease in productivity loss through ADA administration to Japanese RA patients was estimated to be $3.76 billion [28]. 

## 4. Discussion

The socioeconomic impact of RA is substantial, with work disability rate several fold greater than in the general population [51]. Longitudinal data supports the early and progressive impact of RA on work disability over time [52]. Benucci et al. recently discussed the changing structure of RA costs in Italy and how indirect costs consideration is essential for an adequate view of economic disease burden [39]. Furneri et al. indicated that introduction of biologics has significantly raised direct medical costs in a proportion of patients, but at the same time they significantly reduce parameters of disease severity and productivity loss [42]. Trends in RA, for which disability pension has declined in Sweden, may be attributed not only to biologics, but also to more effective treat-to-target strategies. Moreover, a variety of political and demographic factors may also exert a certain effect [53]. Indirect costs as a component of total spending vary by country [54]. The introduction of biosimilars has led to a decrease in public reimbursement, which may lead to greater patient accessibility and more widespread use [55]. Together, these findings substantiate a re-evaluation of the current evidence on biological agent impact on indirect costs, which comprise a major part of RA-related economic burden.

The data analyzed in the current systematic review suggests that biological agents, namely TNF-antagonists, improve indirect costs pertaining to absenteeism and presenteeism measures, which is associated with a varying degree of economic benefit [22,23,24,25,26,28]. Many factors may confound the findings of individual studies, e.g., population characteristics, cost calculation method, and measuring instrument, in which none is ideal for quantification of productivity loss [14]. Several instruments are utilized to measure productivity outcomes, however, although findings are usually skewed into one direction, the magnitude of the effect may differ [23]. The data support beneficial economic effects associated with TNF inhibitor effects on absenteeism in RA, particularly at its early stages, though inter-study heterogeneity in populations and outcome measures limits conclusions in general. In 2006, a systematic review of RA impact on workplace productivity reported that scarce data are available for presenteeism [18], which has changed in recent years. However, less reliable data are available for presenteeism and associated economic gains, but they suggest a beneficial effect incurred by biologics. Aside from the lack of standardization, there are other sources of bias to consider. Questionnaire studies are prone to recalland misattribution bias. Baseline rather than head-to-head comparison, substantial missing data or loss to follow-up, and imputation of presenteeism are other potential confounders.

We identified several reviews in our study. Most recently, Verstappen et al. provided an in-depth overview of studies investigating absenteeism and presenteeism, though without an economic focus. The authors discussed that many studies include longstanding RA, in which patients often experienced work loss at inclusion, which may imply a lesser benefit under biological treatment. Work impairment, absenteeism, and work loss were outlined to share a close relationship, where the focus on only one measure of productivity may obscure clinical and economic evaluation. The authors shortly discussed that sick leave, particularly in early RA, may be improved by biologics, though the data for presenteeism is limited [46]. It was also concluded that the economic gain for society and the employer requires further investigation. Data obtained in this systematical review is in line with these conclusions, though it extends these findings to economic evaluation. An earlier systematic review of similar scope on work participation, conducted in 2011, determined that no pooled effect size can be analyzed due to heterogeneity of data [56]. In line with our results, an overall small positive effect on absenteeism and presenteeism depending on the study comparator was reported for almost all of the included studies, though economic projection was not focused upon.

The majority of studies utilize the HCM [23,24,26,28], which carries some inherent weaknesses. However, the FCM also requires consideration of employee replaceability and appropriate friction period. Both approaches may not be easily generalizable. Zhang et al. emphasized that tailoring to individual work and workplace characteristics would improve the projections [57]. In the BeSt trial analyses, the feasibility of initial IFN combination depended on the method of calculation [38]. The value of projections associated with productivity is dependent on the local healthcare system, and how it attributes productivity. Differences of even several fold magnitude can be observed with varying calculation approaches; as follows from a comprehensive review of RA costs in 2009, which reported loss of productivity amounted to €8452 using HCM compared to €1441 using FCM [58]. Estimated costs per patient and year in Europe were €15,000 and €3800 in Western and Central/Eastern Europe respectively (2008), which underscores the individual healthcare system perspective [40,58]. In another review by Lundkvist et al., indirect costs were €16.584 billion per year in Europe and €8.716 billion per year in the US. Per patient annual values were quite similar: €4300 in Europe and €4400 in the US [59]. In has been discussed that economic analyses also differ in model type and feature, with a subsequent variability in estimates [45]. Prior studies have reported that economic analyses for RA are scarce, whereas indirect costs are a major contributor and their exclusion is a substantial shortcoming [43,45]. With a difficulty in direct comparison, applicability to local healthcare systems is limited [45]. In summary, depending on the method of calculation and comparative scenario, biological agents may compensate expenditures. 

Drug acquisition costs may be influenced by the introduction of biosimilars on the market and associated price erosion, which may alter the contribution of productivity savings to total costs. Cross country inequities in biological access are still prevalent [60], while the distribution of indirect costs also differs by healthcare [54]. Studies from countries with strict reimbursement and low availability of biological agents may report minor improvements in work productivity. This may refer to patients with advanced disease stages where irreversible disability has occurred, which may obscure the beneficial effect of biologics [40]. Some biological drugs have multiple indications in immune mediated inflammatory diseases, which may also improve their cost-efficiency in comorbidity [41]. Fautrel et al. have outlined that although biologics substantially impact work participation, the benefits in patients with more severe and disabling disease are less [40], which unfortunately concerns a considerable proportion of patients encountered in routine practice. Both real-life and trial data are therefore important for decision makers and clinicians alike.

It is necessary to comment on the weaknesses of our study. Incomplete retrieval of records could have occurred due to the adoption of several brand names for biological agents into our search strategy, which may be restricted in certain countries. Comorbidity is prevalent among RA patients, while RA is not the only condition that impacts workplace productivity. Recent reports show that cardiovascular and mental health conditions are among the most common among RA sufferers, in the case of the former they may even convey a difficult-to-control disease profile, while both have been associated with presenteeism, and pose as confounders to our findings [20,61,62]. Depression has also been previously discussed as a particularly difficult confounder when considering productivity outcomes [63]. Moreover, other than DMARD regimens, self-management and non-pharmacological strategies may also impact productivity, which was also not considered in our analyses. We conducted only an analysis of the MEDLINE database, which may not include all relevant reports, while records were considered only in English language, which lead to an exclusion of one study. Furthermore, it should be underlined that the simultaneous use of medicines and effective self-management programs by patients may also affect the results of this study. 

## 5. Conclusions

The burden of RA from both a societal and economic standpoint is high. In the majority of studies, data suggests biological treatment may lead to improved absenteeism and presenteeism, though the magnitude of the effect is variable. Associated monetary savings incurred by biologics are reliant on RA patients retaining employment and work ability. There are several other confounders to the conclusions that have to be kept in mind. High prevalence of comorbidities among RA sufferers makes the attribution of workplace impairment to a sole condition difficult, and therefore indirect economic gains associated with biological therapy are arguably tentative. Delineating the impact of RA alone on absenteeism and presenteeism remains particularly difficult with some of the more commonly co-occurring conditions, i.e., depression, which have been previously identified as confounders. Moreover, self-management strategies other than medication with DMARDs may positively influence workplace productivity, and should also be investigated. Real-life data and head-to-head comparisons comparing biologics and the current standards of care with regard to indirect costs are warranted. There are different populations, comparators, productivity measures, and cost models across studies, which makes a direct comparison difficult. Response to biological therapy is another factor to account for, which would require adequate predictors to determine utility among particular patient groups. 

## Figures and Tables

**Table 1:** Methodology of trials about the effect of biological treatment on absenteeism and presenteeism.

Author	Study Design	Treatment	*n*	Stage of RA	Age Range (Mean)	Disease Duration (Mean)	Absenteeism Measure	Presenteeism Measure	Method	Time Points
	**RCT Trials**									
Allaart 2007 [22]	RCT (BeST)	1. Seq. monotherapy	508	Early RA (ACR criteria)	≥18 (ND)	ND	Three-monthly diary on work absenteeism	-	FCM	Baseline until 2 years
		2. Step-up comb. Therapy (INF) ^c^								
		3. Initial comb. Therapy (INF) ^d^								
		4. MTX + INF ^d^								
Anis 2009 [23]	RCT (COMET)	MTX	100	Early RA (ACR criteria)	≥18 (45.1)	8.9 months	Number of missed work days/WPAI	Reduced working time (in days)/WLQ	HCM	0 and 12 months (weeks 12, 24, 36, 52)
		ETA + MTX	105		≥18 (45.4)	8.6 months	Number of stopped work days ^f^/WLQ			
	**Observational Studies**									
Zhang 2008 [24]	Open-label, multicenter, phase IIIb study (CanAct)	ADA	389	Moderate to severe active RA (ACR criteria)	(55.0)	12.5 years	Number of absent work days multiplied by the individual’s daily wage	Number of extra work hours patients needed to catch up on tasks they were unable to complete during normal working hours multiplied by the individual’s hourly wage	HCM	Baseline and 12 months
Augustsson 2010 [25]	Observational (STURE register)	Anti-TNF (ETA, INF, ADA)	594	ND	18–55 years (40.0)	9.4 years	-	Hours worked/week	ND	Baseline, 6 months, 1, 2, 3, 4, and 5 years
Hone 2013 [26]	Prospective, observational study	ETA	204	Moderate to severe RA	20–67 (46.6)	5.1 years	WPAI measures of absenteeism – work time missed	WPAI measures of presenteeism – impairment at work	HCM	6 months
Klimes 2014 [27]	Bottom-up cross-sectional cost-of-illness study	Without biologics	137	ND	18–64 years (58.9)	13.6 years	Days spent on sick leave, and the period of time spent on full disability pension or partial disability pension	-	FCM	6 months
		With biologics	124		18–64 years (53.6)	15.5 years				
		DMARDs	130		(53.7)	10.1 months				
Tanaka 2018 [28]	Non-interventional trial for up-verified effects and utility (ANOUVEAU) study	ADA ^i^	1 196	Greater portion of the patients had established RA, with moderate disease activity	PW: (50.0)	5.6 years	WPAI measures of absenteeism—work time missed	WPAI measures of presenteeism—impairment at work	HCM	48 weeks

CZP—certolizumab pegol, ADA—adalimumab, DMARD—disease modifying antirheumatic agent, ETA—etanercept, IFX—infliximab, MTX—methotrexate, RCT —randomized clinical trial, ND—no data, NA—not applicable, OWI—overall work impairment, AI—activity impairment, RTX—rituximab, PW—paid worker employed for ≥35 h/week; PTW—part time worker employed for <35 h/week; HM—home maker non-employed; HCM—human capital method, FCM—friction cost method; ACR—American College of Rheumatology; RA—rheumatoid arthritis; WPAWork Productivity and Activity Impairment; (a) to be eligible for treatment with infliximab or etanercept, patients had to have a diagnosis of RA according to clinical judgment and have failed to respond to, or to be intolerant of, at least two DMARDs, including methotrexate; (b) MTX, next steps sulfasalazine, leflunomide, MTX + infliximab, gold, MTX + cyclosporine + prednisone, azathioprine + prednisone; (c) MTX, next steps add sulfasalazine, then hydroxychloroquine, then prednisone, next switch to MTX + infliximab, MTX + cyclosporine + prednisone, leflunomide, gold, azathioprine + prednisone; (d) starting with MTX + sulfasalazine + a tapered high dose of prednisone, next step MTX + cyclosporine + prednisone, next MTX + infliximab, leflunomide, gold, azathioprine + prednisone; (e) starting with MTX + infliximab, next steps sulfasalazine, eflunomide, MTX + cyclosporine + prednisone, gold, azathioprine + prednisone; (f) using mapping methods; (g) the COBRA-light strategy comprises high-dose methotrexate (25 mg/day), combined with medium-dose prednisolone (30 mg/day, tapered to 7.5 mg/day); (h) it comprises a combination of low-dose methotrexate (7.5 mg/day) and sulfasalazine (2 g/day) and initial high dose prednisolone (60 mg/day, tapered to 7.5 mg/day); (i) patients were eligible for the study if they had an inadequate response to conventional therapy (e.g., conventional DMARDs or biologics other than ADA) as stated in the current Japanese labelling recommendations for ADA and met the Japanese guidelines issued by the Japan College of Rheumatology for the use of TNF inhibitors

**Table 2:** Effect of biological treatment on absenteeism studies.

Author	Measure	Comparator	Difference	∆	95%CI	Significant or Not	Costs
**RCT Trials**							
Anis 2009 [23]	Missed work days	MTX vs. ETA+MTX	31.9 vs. 14.2	−17.6	(–34.4; –2.2)	YES	–£1244 ^c,f^
	Reduced working time		19.8 vs. 10.5	–9.3	(–21.9; 3.9)	NO	–£657 ^c,f^
	Stopped worked days		Scenario Ic: 32.9 vs.10.9 Scenario IId: 12.3 vs. 4.8	–22.1 –7.4	(–45.2; –0.3) (–15.9; 1.2)	YES NO	Scenario I: –£1562 ^c,d,f^ Scenario II: –£523 ^c,e,f^
	Total absenteeism		Scenario Ic: 65.6 vs.29.0 Scenario IId: 44.3 vs. 22.3	−36.6 −22.0	(−68.3; −5.9) (−42.6; –2.1)	YES YES	Scenario I: −£2586 ^c,d,f^ Scenario II: −£1555 ^c,e,f^
Allaart 2007 [21]	Overall		Decrease of 0.1 on utility associated with decrease of 2 working h/week			Using the friction-cost method, overall societal costs were estimated at €19,905, €15,926, €17,810, and €28,547 (*p* ≤ 0.05 Group 4 vs. Groups 1–3). Indirect costs: €9113, €8638, €10,001, and €4786 (Groups 1, 2, 3, and 4 respectively) ^a^	
	4. MTX + INF		Productivity was highest in this group				
**Observational Trials**							
Zhang 2008 [24]	Absenteeism, mean	ADA vs. baseline	ND	ND	ND	ND	Lost productivity costs, past two weeks: –$57.21 (mean)
Hone 2013 [26]	Hours gained/patient (absenteeism)	ETA baseline vs. 6 months	71.1 vs. 63.5/ 9.9 ± 20.1 vs. 4.4 ± 16.1	7.6/ −3.5 ± 17.0	ND/ (−6.1; −1.0)	ND/ YES	Economic gain/patient: $1794
Klimes 2014 [27]	Productivity costs	Without vs. with biological treatment	ND	ND	ND	ND	Friction cost approach: €1304 vs. €2090
Tanaka 2018 [28]	Absenteeism	Baseline vs. week 48	ND	ND	ND	ND	Human capital method (cumulative reduction): PW: $9278 (mean) PTW: $6480 (mean) HM: $5449 (mean)

(a) ADA—adalimumab, DMARD—disease modifying antirheumatic agent, ETA—etanercept, IFX—infliximab, MTX—methotrexate, PW—paid worker employed for ≥35 h/week; PTW—part-time worker employed for <35 h/week; HM—home maker non-employed; baseline and 12 months’ status for the entire cohort, extrapolated to annual costs. Work capacity is expressed as full time equivalent—that is, full time work represents 100%, part time work actual percentage, and not working 0%; (b) adjustment for sex, age, HAQ, DAS28, and pain at baseline; (c) negative difference means improvement in productivity or cost savings for ETA + MTX vs. MTX; (d) under Scenario I, the total annual absenteeism was 29 work days for the ETA + MTX group compared with 66 work days for the MTX group. This corresponded to 37 fewer work days lost (95%CI: 6; 68) equaling £2586 productivity gain for the ETA + MTX group; (e) under Scenario II, 22 work days were lost for the ETA + MTX group vs. 44 work days in the MTX groups, resulting in 22 fewer work days lost (95%CI: 2; 43) or a productivity gain of £1555 for the ETA + MTX group; (f) costs = number of days median daily pay in UK weighted by the distribution of work status and sex in COMET data (£70,66).

**Table 3:** Effect of biological treatment on presenteeism studies.

Author	Measure	Comparator	Difference	∆	95%CI	Significant or Not	Costs
**RCT Trial**							
Anis 2009 [23]	WPAI: Work productivity loss at work (%)	MTX vs. ETA + MTX	23.1 vs. 15.6	−7.5	(−11.2; −4.2)	YES	NA
	WPAI: Lost work days due to presenteeism		Scenario I: 34.0 vs. 28.6 Scenario II: 38.9 vs. 29.7	−5.4 −9.3	(−13.5; 2.8) (−16.3; −2.5)	NO YES	−£382 −£657
	WPAI: Total work productivity loss, days		Scenario I: 99.6 vs. 57.6 Scenario II: 83.3 vs. 51.9	−42.0 −31.3	(−69.0; −15.7) (−50.2; −12.6)	YES YES	−£2968 −£2212
	WLQ: work productivity loss at work (%)		6.2 vs. 4.8	−1.4	(−2.1; −0.7)	YES	NA
	WLQ: Lost work days due to presenteeism		Scenario I: 9.1 vs. 8.9 Scenario II: 10.4 vs. 9.2	−0.3 −1.3	(−2.3; 1.8) (−2.8; 0.3)	NO NO	−£21 −£92
	WLQ: Total work productivity loss, days		Scenario I: 74.7 vs. 37.8 Scenario II: 54.8 vs. 31.5	−36.9 −23.3	(−66.9; −7.6) (−43.0; −4.2)	YES YES	−£2607 −£1646
**Observational trials**							
Zhang 2008 [24]	Absenteeism, mean	ADA vs. baseline	ND	ND	ND	ND	Lost productivity costs, past two weeks: –$4.48
Augustsson 2010 [25]	Overall	Unadjusted model	Improvement: first year: 4.2 h/week, thereafter: 0.5 h/week			The productivity gains for society in patients continuing treatment would total €28,000 over 5 years.	Note that these estimates only apply to patients who do not discontinue treatment, a group that may be difficult to identify before treatment initiation.
		Adjusted model ^b^	Improvement: first year: 4.1 h/week, thereafter: no change			The productivity gains for society in patients continuing treatment would total €27,000 over 5 years. This corresponds to approximately 40% of the annual anti-TNF drug cost.	
Hone 2013 [26]	Hours gained/patient (presenteeism)	ETA baseline vs. 6 months	205.2 vs. 189.7/ 39.7 ± 24.5 vs. 24.8 ± 22.5	15.5/ −13.5 ± 23.3	ND/ (−17.0; −9.9)	ND/ YES	Economic gain/patient: $5328
Tanaka 2018 [28]	Presenteeism	Baseline vs. week 48	ND	ND	ND	ND	Human capital method (cumulative reduction): PW: $5836 (mean) PTW: $2726 (mean) HM: NA

ADA—adalimumab, DMARD—disease modifying antirheumatic agent, ETA—etanercept, IFX—infliximab, MTX—methotrexate, PW—paid worker employed for ≥35 h/week; PTW—part-time worker employed for <35 h/week; HM—home maker non-employed; WLQ—Work Limitations Questionnaire; (a) Baseline and 12 months’ status for the entire cohort, extrapolated to annual costs. Work capacity is expressed as full time equivalent—that is, full time work represents 100%, part time work actual percentage, and not working 0%; (b) own estimation based on data presented in publication; (c) using the friction cost method for valuation of productivity losses, the infliximab group had borderline higher productivity losses (€14,597 vs. €12,018; adjusted mean difference €2134; 95%CI: −284; 4535), and (as with the human capital method) higher total costs (€42,084 vs. €22,382; adjusted mean difference €19,090; 95%CI: 15,564; 22,252) than the conventional treatment group; DMARD—disease-modifying antirheumatic drug.

## Appendix A

**Table A1:** Search strategy.

N.	Query	Items Found
#1	adalimumab* OR Humira OR (D2E7 AND antibody) OR adalimumab [MeSH]	6987
#2	etanercept* OR Enbrel OR Erelzi OR Benepali OR (TNR–001 AND "fusion protein") OR etanercept [MeSH]	7804
#3	tocilizumab* OR RoActemra OR atlizumab OR tocilizumab [MeSH]	2380
#4	certolizumab* OR Cimzia OR (CDP870 OR CDP–870) OR certolizumab [MeSH]	1072
#5	rituximab* OR MabThera OR rituximab [MeSH]	19,933
#6	anakinra OR Kineret OR anakinra [Mesh]	5464
#7	abatacept OR Orencia OR abatacept [MeSH]	3353
#8	infliximab OR Remicade OR infliximab [MeSH]	13,165
#9	golimumab OR Simponi OR golimumab [MeSH]	962
#10	biologic* OR bio-logic* OR "bio logic*" OR biosimilar* OR bio-similar* or "bio similar*"	1,735,869
#11	#1 OR #2 OR #3 OR #4 OR #5 OR #6 OR #7 OR #8 OR #9 OR #10	1,776,784
#12	"rheumatoid arthritis" OR RA	159,750
#13	#11 AND #12	19,542
#14	(Indirect OR Productivity) AND (Cost OR Costs OR Cost* OR (Human AND Capital))	64,023
#15	absenteeism OR presenteeism	11,562
#16	"human capital method"" OR HCM OR ""willingness to pay method"" OR WTP OR ""friction cost method"" OR FCM	15,253
#17	#13 AND (#14 OR #15 OR #16)	153

**Table A2:** Risk of bias in randomized controlled trials included in systematic review.

Domain	BeST Allaart 2007 [22]	COMET Anis 2009 [23]
Random sequence generation	Unclear	Low
Allocation concealment	Unclear	Low
Blinding (participants and personnel)	High	Low
Blinding (outcome assessment)	Low	Low
Incomplete outcome data	Unclear	Unclear
Selective reporting	High	Low
Other sources of bias	Unclear	Low

**Table A3:** Risk of bias in observational trials included in systematic review.

Quality Assessment	Zhang 2008 [24]	Augustsson 2010 [25]	Hone 2013 [26]	Klimes 2014 [27]	Tanaka 2018 [28]
Case series collected in more than one centre, i.e., multi-center study	Yes	Yes	Yes	No	Yes
Is the hypothesis/aim/objective of the study clearly described?	Yes	Yes	Yes	No	Yes
Are the inclusion and exclusion criteria (case definition) clearly reported?	Yes/No	Yes/No	Yes	No	Yes
Is there a clear definition of the outcomes reported?	Yes	Yes	Yes	Yes	Yes
Were data collected prospectively?	Yes	Yes	Yes	No	Yes
Is there an explicit statement that patients were recruited consecutively?	Yes	Yes	Yes	Yes	Yes
Are the main findings of the study clearly described?	Yes	Yes	Yes	Yes	Yes
Are outcomes stratified? (e.g., by disease stage, abnormal test results, patient characteristics)	Yes	Yes	Yes	Yes	Yes
